# *Entamoeba histolytica* Cysteine Proteinase 5 Evokes Mucin Exocytosis from Colonic Goblet Cells via αvβ3 Integrin

**DOI:** 10.1371/journal.ppat.1005579

**Published:** 2016-04-13

**Authors:** Steve Cornick, France Moreau, Kris Chadee

**Affiliations:** Department of Microbiology, Immunology and Infectious Diseases, Snyder Institute for Chronic Diseases, University of Calgary, Calgary, Alberta, Canada; University of Virginia Health System, UNITED STATES

## Abstract

Critical to the pathogenesis of intestinal amebiasis, *Entamoeba histolytica* (*Eh*) induces mucus hypersecretion and degrades the colonic mucus layer at the site of invasion. The parasite component(s) responsible for hypersecretion are poorly defined, as are regulators of mucin secretion within the host. In this study, we have identified the key virulence factor in live *Eh* that elicits the fast release of mucin by goblets cells as cysteine protease 5 (*Eh*CP5) whereas, modest mucus secretion occurred with secreted soluble *Eh*CP5 and recombinant CP5. Coupling of *Eh*CP5-αvβ3 integrin on goblet cells facilitated outside-in signaling by activating SRC family kinases (SFK) and focal adhesion kinase that resulted in the activation/phosphorlyation of PI3K at the site of *Eh* contact and production of PIP3. PKCδ was activated at the *Eh*CP5-αvβ3 integrin contact site that specifically regulated mucin secretion though the trafficking vesicle marker myristoylated alanine-rich C-kinase substrate (MARCKS). This study has identified that *Eh*CP5 coupling with goblet cell αvβ3 receptors can initiate a signal cascade involving PI3K, PKCδ and MARCKS to drive mucin secretion from goblet cells critical in disease pathogenesis.

## Author Summary

An interesting facet to the protozoan parasite *Entamoeba histolytica* is the ability to cause disease in a very limited subset of individuals, subject to first overcoming the intestinal mucus barrier within the gastrointestinal tract. Mucins, which are the primary constituent of the mucus layer are secreted basally to maintain the barrier and also in response to a variety of pathogens and noxious threats to protect the sensitive epithelium. Unfortunately, the mechanisms and signal cascades that regulate this secretion event are largely unknown. Here we describe how one such pathogen targets a specific host receptor on mucin-secreted cells to elicit secretion by activating distinct signaling pathways. Further, we have identified the parasite component responsible for this event. Our study provides insight in the pathogenesis of *E*. *histolytica* along laying the foundation for a broader understanding of how mucin secretion is regulated. We believe the pathways and mechanisms identified here can be applied to a wide-array of pathogens to understand how pathogens are kept away from the epithelium and how exploitation of this may lead to disease.

## Introduction

The secreted polymeric mucin layer that lies above the host epithelium forms the first line of innate host defense within the gastrointestinal tract [1]. Secreted mucus was recently characterized to have bimodal phases, with an inner firmly sterile adherent layer and an outer loosely adherent layer that serves as the primary colonization area for microbes in the gut [2]. The principal mucin present in the colonic mucus layer is MUC2, a heavily glycosylated protein composed of a 5179 amino acid backbone and mostly O-linked sugars [3–5]. This glycosylation is predominantly focused within the variable tandem repeat domains in the central core of the molecule at serine/threonine residues whereby N-acetylgalactosamine is the first core 3 branched sugar [6]. MUC2 is mainly composed of galactose, N-acetylgalactosamine, N-acetylglucosamine with terminal fucose and sialic acid residues that are often targeted by microbes via adherence lectins [7,8]. It is likely these sugar moieties present on MUC2 act as decoys to keep the indigenous microbiota and pathogenic organisms spatially separated from the host epithelium [1].

Several enteric pathogens have adapted mechanisms to overcome the mucus barrier by targeting MUC2 for degradation [1,9,10]. One such pathogen is the protozoan parasite *E*. *histolytica (Eh)*, which is responsible for amebiasis and substantial morbidity in developing countries [11]. Interestingly in the vast majority of individuals, *Eh* colonization is restricted to the intestinal lumen and outer mucus layer resulting in asymptomatic infections. *Eh* binds with high affinity to MUC2 mucin via a 170kDa heavy subunit adherence lectin that specifically targets Gal/GalNAc side chains [12,13]. In the absence of a mucus barrier, *Eh* uses the Gal/GalNAc lectin to bind host cells and to induce cytolysis [14]. In mice lacking a bona fide mucus barrier (*Muc2* ^*-/-*^), *Eh* induces a potent pro-inflammatory and secretory response with loss of barrier integrity [15]. In the presence of a mucus barrier, *Eh* cysteine proteinase 5 (*Eh*CP5) target the less glycosylated C-terminus of MUC2 for cleavage [16]. This leads to disruption of the mucus gel and loss of its protective functions [17]. Inherently, this allows *Eh* to make contact with the host epithelium and to induce pro-inflammatory responses and epithelial cell disruption. In opposition of this, goblet cells can mount a robust hyper secretory response to repel invading pathogen and noxious substances [1,18]. While effective to some degree, sustained hypersecretion of mucus leads to depletion of mucin stores due to a slow turnover rate [3]. In *Eh* infection, this leaves the epithelium vulnerable for *Eh* contact with epithelial cells leading to contact-dependent cytolysis in disease pathogenesis.

How intestinal goblet cells release mucin constitutively and in response to pathogens is still unclear and beyond signaling cascades that modulate transcription of MUC2, very little is known on how kinases modulate mucus secretion. In *Eh* infections, this event was characterized to be contact-dependent and inhibited by the addition of exogenous galactose [19]. In this study, we have unraveled that secreted and membrane bound *Eh*CP5 is a potent mucus secretagogue. Specifically we show that *Eh*CP5 coupling to αvβ3 integrin receptors on goblet cells stimulated Src family kinases and PI3K signaling event. This culminates in PKCδ activation whereby through phosphorylation of the vesicle trafficking protein myristoylated alanine-rich C-kinase substrate (MARCKS), stimulates robust and sustained mucin exocytosis. Thus, in addition to cleaving the C-terminus of MUC2 to abrogate MUC2 protective functions, *Eh*CP5 plays an essential role in contact-dependent mucin hypersecretion during the pathogenesis of intestinal amebiasis.

## Results

### *E*. *histolytica*-induced mucin secretion is cysteine proteinase dependent

**Fig 1:**
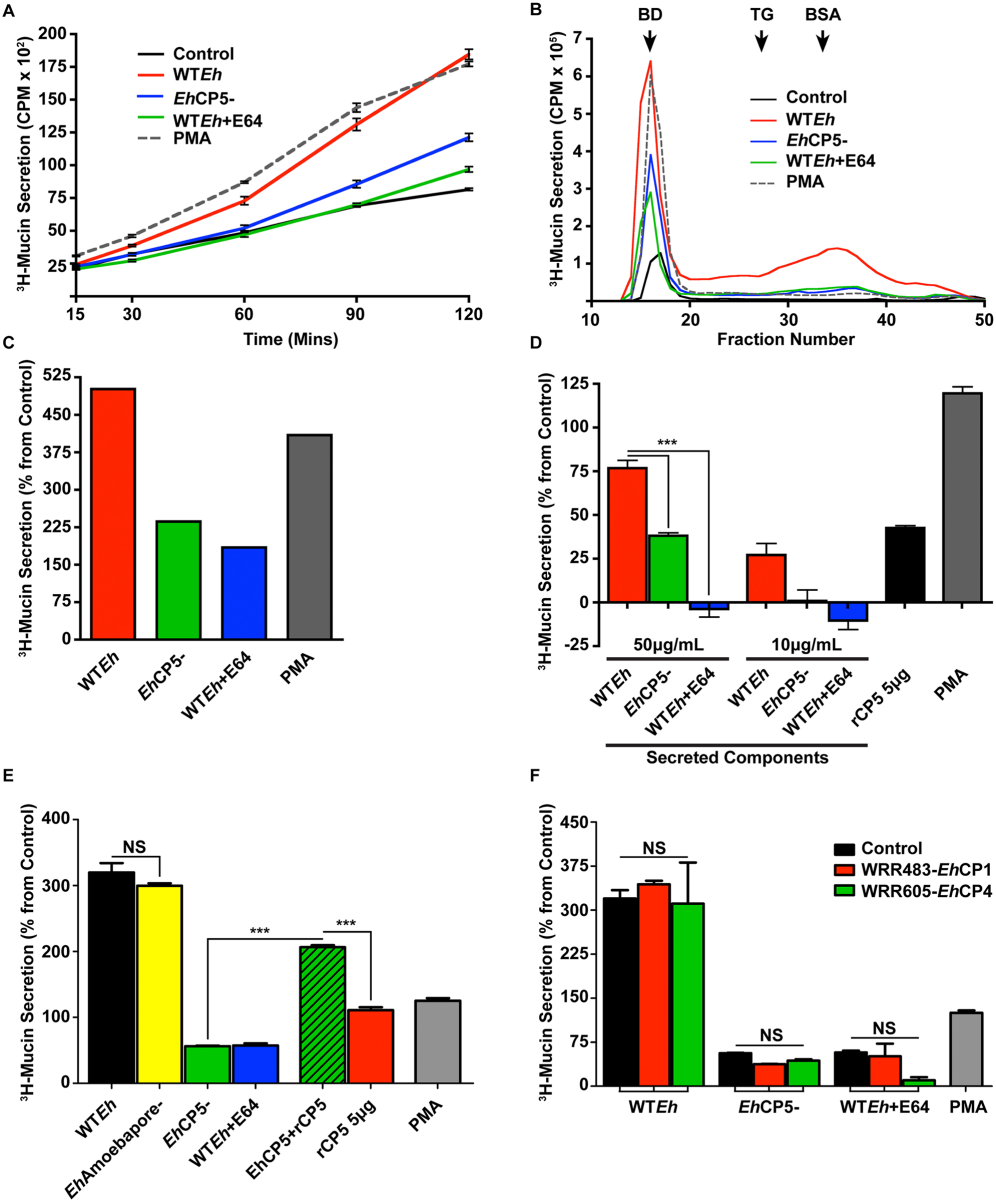
*Entamoeba histolytica* evokes mucin secretion via cysteine protease 5. LS174T colonic cells metabolically labeled with ^3^H-glucosamine were assayed for mucin secretion in response to live WT*Eh*, *Eh*CP5^-^ and WT*Eh* treated with the cysteine protease inhibitor E64. The phorbol-ester PMA (1μM) was used as a positive control to elicit mucin hypersecretion. **A**. Mucin secretion was assessed at various time points with 5 x 10^4^ *Eh*/well. **B**. After 2 h, supernatants containing secreted mucins were precipitated 10% TCA and 1% PTA and loaded onto a Sepharose 4B column. 1mL fractions were collected to assess the molecular weight profile of secreted ^3^H-mucin following *Eh* infection. The column was calibrated with Blue dextran [BD; 2,000,000Da that elutes in the void volume (Vo fractions 16–19) consistent with high molecular weight mucins] and Thyroglobulin (Ty; 669,000Da) and BSA (66,000Da). **C**. Area under the curve was calculated for the Vo of the Sepharose 4B column for each condition and plotted as percent from baseline secretion. **D**. Secreted components (SC) from *Eh* and recombinant CP5 were assayed for their ability to induce mucin secretion in a dose-dependent manner. **E**. Live Amoebapore-deficient *Eh* and *Eh*CP5- complemented with exogenous rCP5 were assayed for their ability to induce mucin secretion. **F.** Specific inhibitors for *Eh*CP1 (WRR483; 20μM) and *Eh*CP4 (WRR605; 20μM) incubated with *Eh* and mucin secretion quantified. ***p <0.001.

To study mucin secretion *in vitro*, the highly proliferative colonic goblet phenotypic cell line LS174T was used. These cells natively produce an abundance of MUC2 mucin and secrete both constitutively and in response to a variety of mucin secretagogues [14]. *Eh* was placed in direct contact with LS174T confluent monolayers at a multiplicity of infection (MOI) of 0.2, a dose determined to be maximal for mucin secretion while not inducing destruction of the monolayer or causing significant cell death (S1A Fig). To determine the kinetics of mucin secretion in response to *Eh*, mucin was metabolically labeled with ^3^H-glucosamine and released in the media were counted in the supernatant following various time points after infection (Fig 1A). WT*Eh* induced robust and fast secretion of mucus comparable to phorbol-ester PMA, a potent mucus secretagogue that activates protein kinase C (PKC) [20]. In contrast, *Eh* silenced for cysteine protease 5 (*Eh*CP5^-^) were unable to mount a secretory response comparable to WT*Eh*. Thus, to determine if cysteine protease activity was involved in mucin secretion, WT*Eh* were pretreated with the cysteine protease inhibitor E64. As predicted, ^3^H-mucin secretion from WT*Eh* + E64 was significantly less than WT*Eh* and was similar to *Eh*CP5^-^. To quantify if *Eh*-induced mucin and non-mucin glycoprotein secretion, supernatants from ^3^H-labeled LS174T cells were collected and analyzed by Sepharose 4B size exclusion chromatography. The majority of the ^3^H-labeled glycoproteins released in response to WT*Eh* and PMA were high molecular weight mucin eluted in the void volume [Vo fractions 16–19 determined by blue dextran (BD) elution; Fig 1B) and low molecular weight glycoproteins that were 3-fold less abundant than high molecular weight mucins [14]. The area under the curve for Vo mucin (Fig 1C) showed that WT*Eh* induced 500% increase in mucin secretion over controls and was even higher than the positive mucin secretagogue PMA. As expected, *Eh*CP5^-^ and WT*Eh* + E64 stimulated a negligible amount of mucin. Viability of *Eh* was a requirement for inducing mucin secretion as glutaraldehyde fixed or heat killed *Eh* did not evoke mucin secretion (S1B Fig). The discrepancy in mucin secretion observed between WT*Eh*, *Eh*CP5^-^ and WT*Eh* + E64 treated cells was confirmed to not be due to an inability to adhere to LS174T cells (S1C Fig). Importantly, WT*Eh*, *Eh*CP5^-^ and WT*Eh* + E64 cells all adhered about 60% to LS174T monolayers after 30 minutes.

To determine if soluble cysteine proteinase could evoke mucin secretion in the absence of live *Eh*, the secreted components (SC) from WT*Eh*, *Eh*CP5^-^ and WT*Eh* + E64 were added to LS174T monolayers (Fig 1D). There was a modest dose-dependent secretion of ^3^H-mucins in response to WT*Eh* SC, a weaker response toward *Eh*CP5^-^ SC and no secretion in response to WT*Eh* + E64 SC. To confirm that *Eh*CP5 was responsible for evoking mucin secretion, recombinant CP5 alone was used and it induced modest mucin secretion. By Western blotting we determined the presence of *Eh*CP5 in both SC and the recombinant protein. WT*Eh* SC contained a weak band for the pro-form of CP5 whereas the majority of immunoreactivity is at 25kDa consistent with the active cleaved form of *Eh*CP5 (S1D Fig). Conversely, *Eh*CP5^-^ SC does not contain either the pro or active forms as expected by the lack of bands at 35- and 25 kDa, respectively. WT*Eh* + E64 SC accumulated pro-CP5 due to the inability for autocatalysis and lack the active 25 kDa cleaved fragment. Recombinant CP5 contains both pro and active CP5. The relative proteinase activity of the SC and recombinant CP5 was tested by Z-Arg-Arg-pNA and as expected, recombinant CP5 and WT*Eh* SC displayed high enzyme activity whereas, *Eh*CP5^-^ SC showed low cysteine proteinase activity (S1E Fig). *Eh*-induced mucin secretion was strictly a post-translational event, as MUC2 mRNA was not up regulated even after 2 h exposure with *Eh* (S1F Fig). This is in contrast to PMA that up regulated MUC2 and MUC5AC mRNA. To exclude any contribution in mucin secretion by the AmoebaporeA gene and to control for the gene silencing technology used to generate *Eh*CP5-, a vector control was used (*Eh*Amoebapore-) that showed no difference as compared to WT*Eh* (Fig 1E). Additionally, *Eh*CP5- that were complemented by adding back exogenous recombinant CP5 restored mucin secretion greater than the sum of just rCP5 or *Eh*CP5- secretion combined demonstrating CP5 acts synergistically with live Eh. The vast majority of mucin secretion appeared to be CP5-dependent as specific inhibitors for two additional *Eh* cysteine proteinases, *Eh*CP1 (WRR483) and *Eh*CP4 (WRR605), failed to inhibit mucin secretion (Fig 1F) [21]. We next interrogated the target of CP5 and its respective protease activity included two classical mucin secretagogues, calcium and ATP. Surprisingly, neither calcium nor ATP was differentially modulated by *Eh*CP5^-^ or when cysteine protease activity was inhibited with E64 as compared to WT*Eh* (S2A and S2B Fig).

### *Eh*CP5 interacts with host αvβ3 integrin

**Fig 2:**
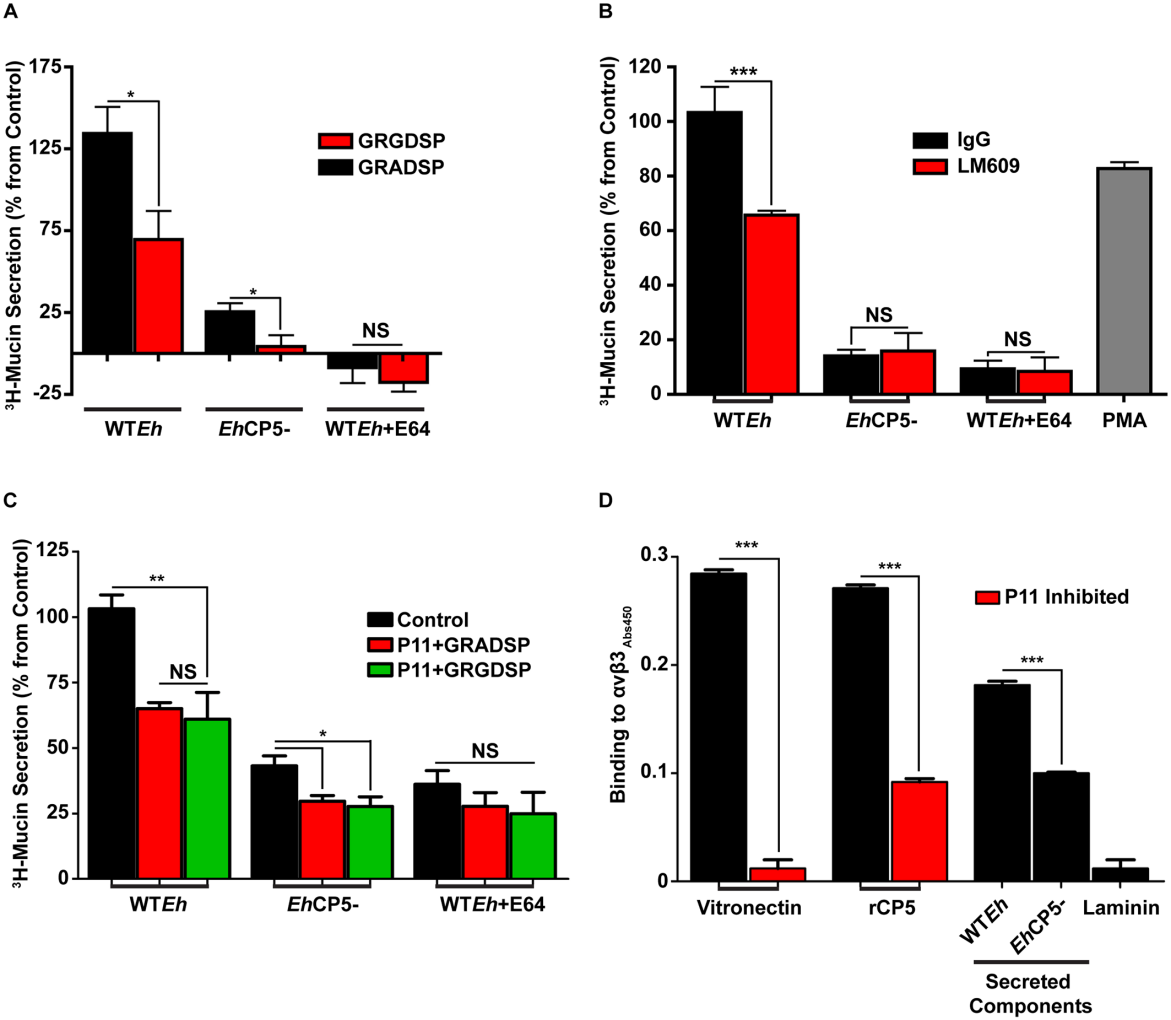
αvβ3 is the critical CP5 receptor on goblet cells responsible for mucin secretion. Mucins from LS174T goblet cells were metabolically labeled with ^3^H-glucosamine and assayed for mucins secretion in the presence of **A**, G**RGD**SP (50μM) and **B**, αvβ3 function-blocking antibody (LM609 5μg/mL) for 2 h with live *Eh*. These were compared to the appropriate controls G**RAD**SP and mouse IgG respectively. **C.** *Eh* induced mucin secretion in the presence of a specific αvβ3 inhibitor P11 (10 μM) with either G**RAD**SP (50μM) or G**RGD**SP (50μM). **D.** αvβ3 binding to various adhesive proteins as assessed by ELISA. Vitronectin and laminin are the positive and negative controls respectively. For P11 inhibition, the inhibitor was included when αvβ3 was added and used at a concentration of 1μM. ***p <0.001, **p <0.01, *p <0.05.

We have previously shown that *Eh*CP5 interacts with host integrin to modulate downstream signaling cascades such as NF-κB and inflammasome activation [22, 23]. *Eh*CP5 contains an RGD binding motif that facilitates this interaction. To investigate if *Eh*CP5-induced mucin secretion was dependent on integrin engagement, small peptide inhibitors of the RGD binding motif present on various integrins were used. Normally these motifs interact with components of the extraceullar matrix such as fibronectin or vitronectin. This acts as a recognition system to mediate critical cellular tasks such as cell adhesion [24]. As predicted, the RGD blocking peptide significantly inhibited mucin secretion from LS174T cells in response to WT*Eh* (Fig 2A). This effect was not observed with WT*Eh* + E64 or with the RAD control peptide. A modest reduction in *Eh*CP5- mucin secretion was observed with the RGD blocking peptide however; overall secretion was low as compared to WT*Eh*. These results were corroborated using function blocking antibodies specific against αvβ3 that markedly reduced WT*Eh* mucin secretion (Fig 2B). Differences in mucin secretion were not observed with *Eh*CP5^-^ or WT*Eh* + E64 or IgG isotype control antibodies. To discern if other RGD-dependent integrin’s have a role in CP5 induced mucin secretion the specific αvβ3 inhibitor P11 was used with either RGD blocking or RAD control peptides. P11 significantly blocked mucin secretion in both WT*Eh* and *Eh*CP5- as compared to control however, no further inhibition was observed when the RGD blocking peptide was also included suggesting αvβ3 is the predominant RGD-dependent integrin that initiates signaling at the cell surface (Fig 2C). An ELISA binding assay was used to assess the αvβ3-CP5 interaction using purified recombinant proteins. Recombinant CP5 bound to αvβ3 at a similar concentration as the positive control vitronectin, which could be inhibited significantly by addition of P11 (Fig 2D). Predictably, less *Eh*CP5- secreted components bound to αvβ3 compared to WT*Eh*.

**Fig 3:**
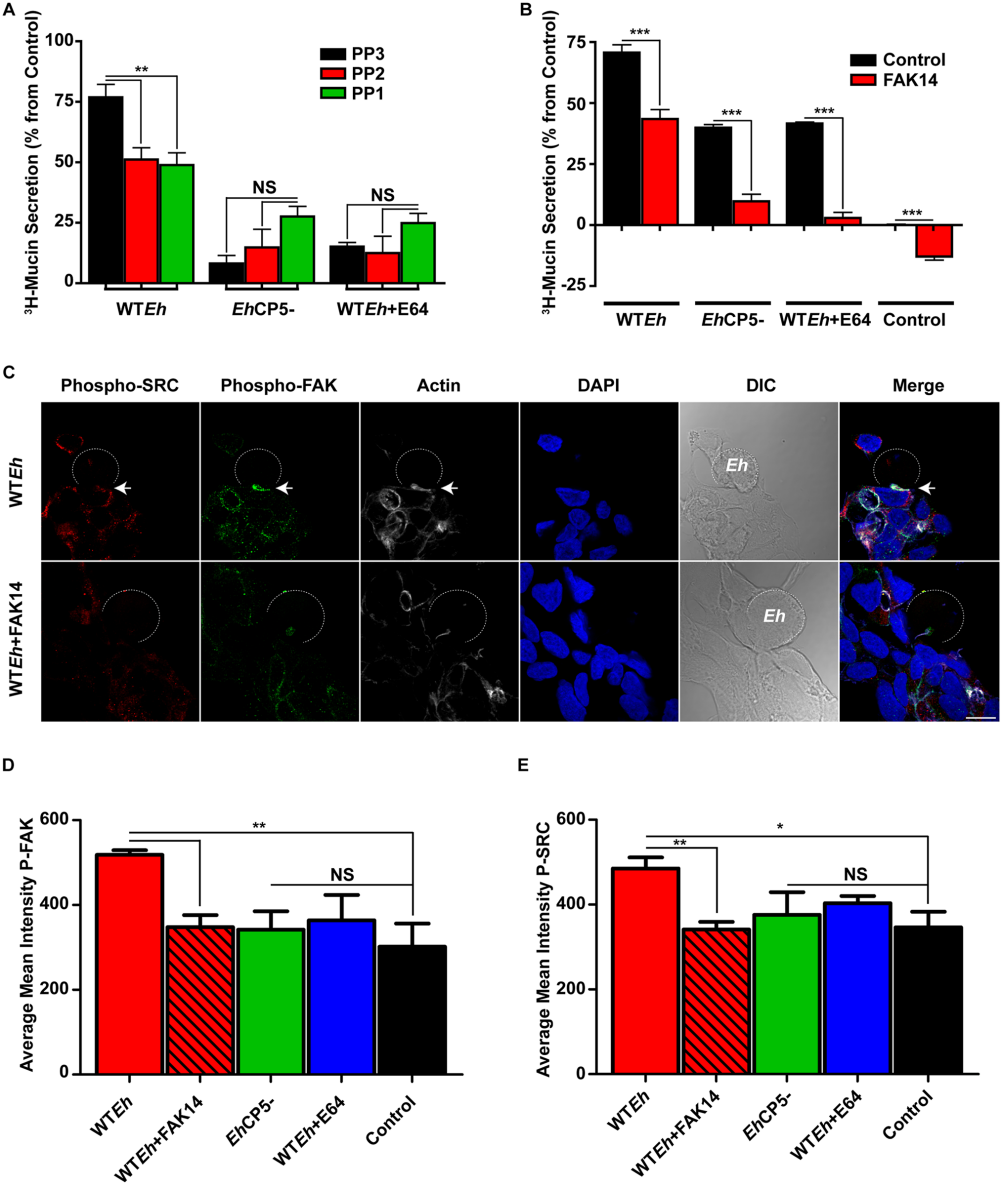
CP5-αvβ3 engagement leads to SRC and FAK activation. **A**. LS174T cells were pre-treated with SRC family kinase inhibitors (PP1 and PP2; 5μM) along with the appropriate control (PP3; 5μm) and assayed for the ability to secrete mucins following metabolic labeling with ^3^H-glucosamine. **B**. The role of FAK in facilitating mucin secretion was assessed by pre-treating LS174T cells with the FAK inhibitor FAK14 (25μM). **C**. LS174T cells were infected with 2x10^5^ *Eh*/well with WT*Eh* in the presence and absence of FAK14 for 20 minutes, then immunostained for phospho-FAK and SRC. The dotted white line indicates the position of *Eh*. The arrows indicate a site of *Eh* contact that induces phosphorylation of SRC and FAK. **D**, **E**. Quantification of confocal images was performed by measuring the concentration of phosphorylated FAK and SRC within the host cell in the immediate contact site by *Eh*. Scale bar 10 μm. ***p <0.001, **p <0.01, *p <0.05.

To assess if *Eh*CP5-αvβ3 binding activated downstream signaling cascades characteristic of outside-in integrin signaling, the SRC inhibitors PP1 and PP2 were employed. These inhibitors specifically target non-receptor tyrosine kinases yet do not affect the activity of EGFR [25]. PP1 and PP2 both displayed the same ability to inhibit mucin secretion in response to WT*Eh* (Fig 3A) whereas the inactive analog PP3 and *Eh*CP5^-^ or WT*Eh* + E64 did not significantly reduce mucin secretion. One of the functions of SRC family kinases in the focal adhesion complex is to facilitate phosphorylation and activation of FAK upon integrin engagement. Treatment of LS174T cells with FAK14 inhibitor negated mucin secretion irrespective of cysteine protease activity or CP5 presence as well as constitutive secretion (Fig 3B). This demonstrates that all forms of mucin secretion are dependent on FAK including constitutive/basal secretion. To assess the phosphorylation status of FAK and SRC, confocal microscopy was used to visualize the interface between goblet cells and *Eh*. Strikingly, FAK and SRC were both observed to be phosphorylated at the site on contact by WT*Eh*, an event that was blocked by pretreatment with the FAK inhibitor FAK14 (Fig 3C). Quantification of phosphorylated SRC and FAK at the *Eh* interface of host cells demonstrated specific activation by WT*Eh* that was inhibited by FAK14 and did not occur with other strains of *Eh* tested (Fig 3D and 3E).

### *Eh*CP5-integrin interaction activates PI3K

**Fig 4:**
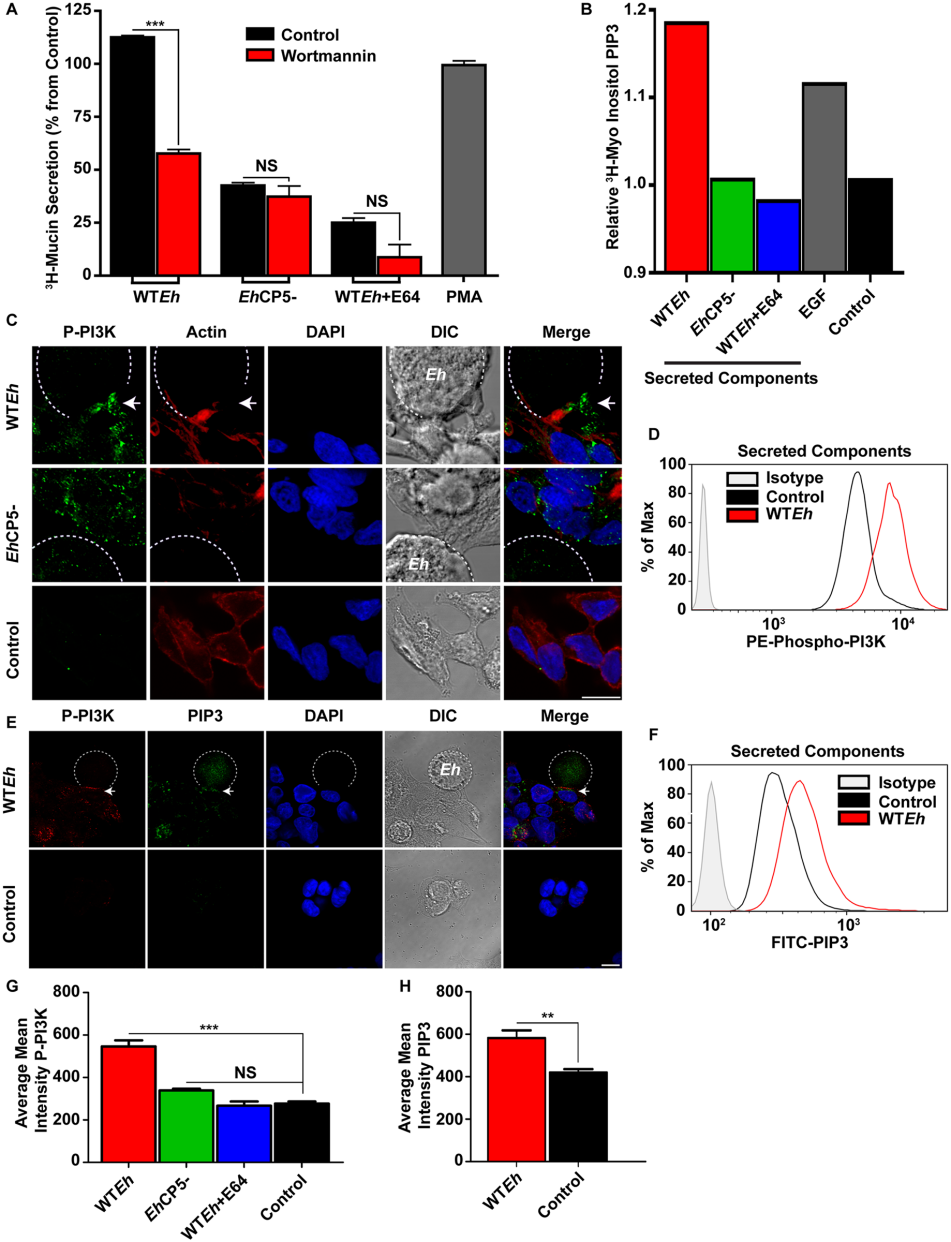
*E*. *histolytica* activates PI3K via CP5 to evoke mucin secretion. **A**. Mucins from LS174T goblet cells metabolically labeled with ^3^H-glucosamine were treated with the PI3K inhibitor wortmannin (10μM) and assayed for secretion of ^3^H-labeled mucins after 2 h in response to *Eh*. **B**. PIP3 accumulation was quantified by counting radiolabeled PIP3 extracted from confluent LS174T monolayers in response to secreted components (50μg/mL) from WT*Eh*, *Eh*CP5^-^, WT*Eh* + E64 and the positive control epidermal growth factor (50ng/mL). Control cells were uninfected basal resting cells. **C**. Confocal microscopy on fixed monolayers infected with WT*Eh* and *Eh*CP5^-^ were imaged with a Phospho-PI3K antibody to detect activation. The dotted white line indicates the position of *Eh*. Scale bar 10 μm. **D**. Flow cytometry analysis of LS174T cells labeled with PE-Phospho-PI3K antibody after stimulation with 50μg/mL WT*Eh* secreted components. As above with Phospho-PI3K, PIP3 was visualized by **E,** confocal microscopy and **F**, Flow cytometry. The PIP3 antibody was routinely observed to strongly cross-react with *Eh* and contact with host cells did not affect this off target staining. Arrows indicate the *Eh*-goblet cell contact site that contains P-PI3K or PIP3 respectively. **G, H.** Quantification of confocal images was performed by measuring the concentration of phosphorylated PI3K and PIP3 within the host cell in the immediate contact site by *Eh*. ***p <0.001, **p <0.01.

Src-family kinase activation acts on a variety of downstream targets one of which is PI3K [26]. To determine if mucin secretion in response to *Eh* was signaling through this pathway, cells were pretreated with PI3K inhibitors wortmannin and Ly294002. WT*Eh* induced mucin secretion was significantly inhibited in cells pretreated with both wortmannin (Fig 4A) and Ly294002 (S3A Fig). In *Eh*CP5^-^ or WT*Eh* + E64, the inhibitors did not significantly reduce mucin release with the exception of *Eh*CP5- following LY294002 treatment which was modestly reduced. While PI3K activation results in downstream signaling to AKT, the functions of AKT were not involved in mucin secretion, as inhibition with triciribine had no effect on secretion (S3B Fig). Upon activation, the regulatory subunits of PI3K (p55/p85) become phosphorylated and PIP2 phosphorylates to PIP3. PIP3 can then act as a potent second messenger to induce activation of various proteins through a pleckstrin homology domain [27]. To confirm PIP3 was generated in an *Eh*CP5-αvβ3-dependent manner, phosphoinositols were metabolically labeled and subsequently pulled down with a specific PIP3-binding protein, GST-GRP1-PH (Fig 4B). WT*Eh* SC induced an increase in PIP3 levels even greater than the positive control epidermal growth factor (EGF) in LS174T cells whereas SC lacking CP5 or cysteine protease activity did not. Confocal microscopy was used to discern the activation status of PI3K in response to live WT*Eh* (Fig 4C). Phosphorylated PI3K was observed at the *Eh*-goblet cell interface in response to live WT*Eh* but not to *Eh*CP5^-^. Interestingly, F-actin was also commonly observed at the interface of contact in response to live WT*Eh*. Flow cytometry was used to quantify the activation state of PI3K following stimulation with WT*Eh* SC (Fig 4D). After 15 minutes stimulation, Phospho-PI3K mean fluorescence intensity was significantly increased over resting cells. PIP3 was localized to the contact site of WT*Eh* and co-localized with Phospho-PI3K as determined by confocal microscopy (Fig 4E). Quantification was performed by flow cytometry using a FITC-PIP3 antibody where WT*Eh* SC induced an increase in mean fluorescence intensity over resting cells (Fig 4F). Abundance of phosphorylated PI3K and PIP3 was quantified from confocal images demonstrating WT*Eh*-induced activation at the contact site was significantly higher than other strains tested (Fig 4G and 4H). Although in some systems PIP3 can directly modulate exocytosis, intestinal goblet cells likely utilize PKC to facilitate mucin exocytosis [28]. Accordingly, PIP3 can interact with PDK1 to directly activate a variety of PKC isoforms. When LS174T cells were treated with the PDK1 inhibitor GSK2334470, all mucin secretion regardless of CP5 or protease activity was inhibited including constitutive secretion (S3C Fig). This likely suggests that all mucin secretion regardless of stimuli is dependent on PDK1 activity.

### *Eh*CP5 induced mucin secretion is PKCδ dependent

**Fig 5:**
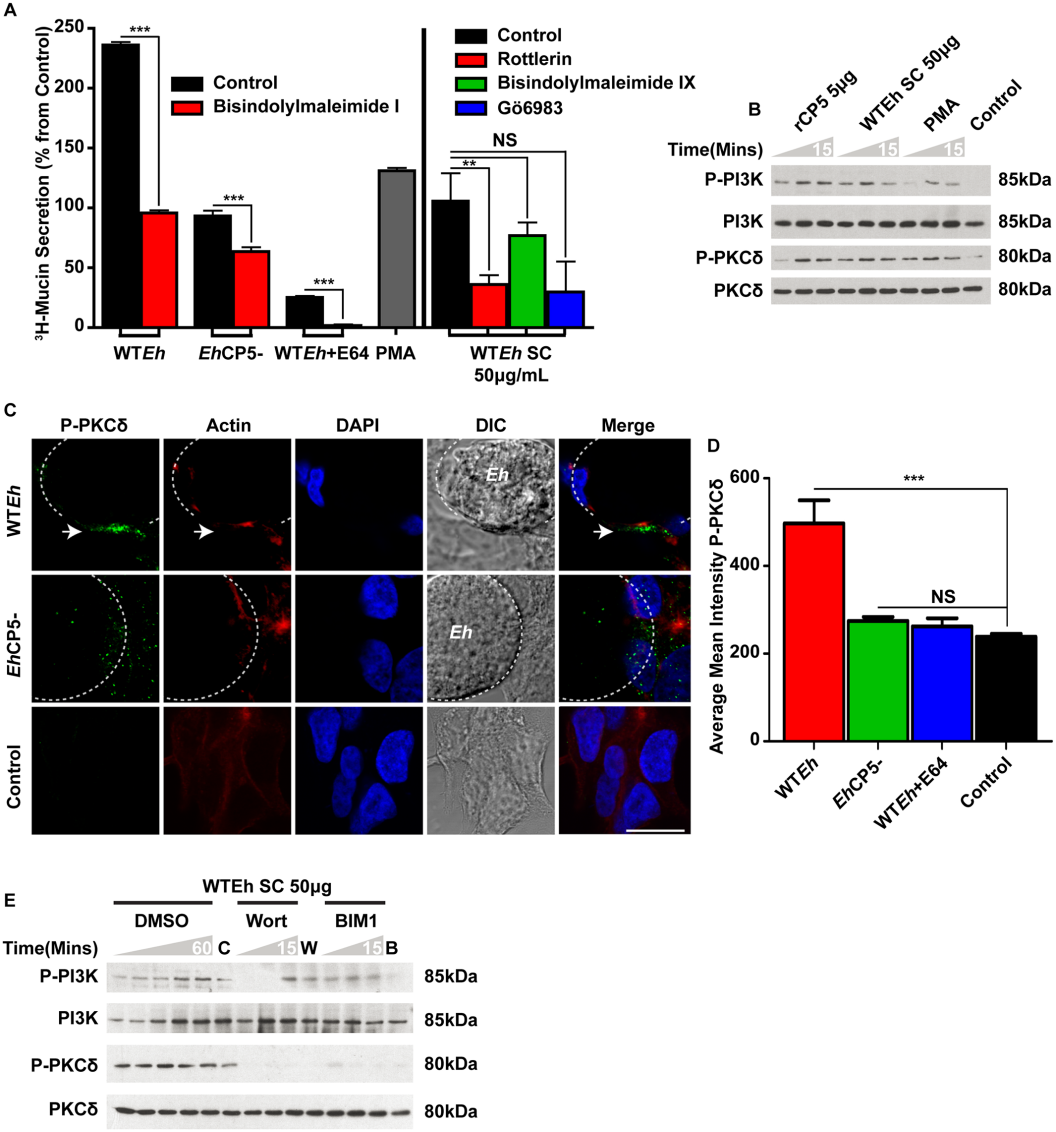
PKCδ is activated downstream of PI3K and is necessary for *Eh*CP5 induced mucin secretion. **A**. Mucins from LS174T goblet cells metabolically labeled with ^3^H-glucosamine were treated with the broad spectrum PKC inhibitor bisindolylmaleimide I (10μM), infected with *Eh* for 2 h and assayed for ^3^H-labeled mucin secretion. A panel of PKC inhibitors including Rottlerin (20μM), Bisindolylmaleimide IX (10μM) and Gö6983 (10μM) was used to discern the critical isoform in response to WT*Eh* secreted components (50μg/mL). **B**. WT*Eh* secreted components (50μg/mL) and recombinant *Eh*CP5 (5μg) were added to LS174T cells and activation of PI3K and PKC determined over a 15-minute period. **C**. Confocal microscopy on fixed monolayers infected with WT*Eh* and *Eh*CP5^-^ were imaged with a Phospho-PKCδ antibody to detect activation. The dotted white line indicates the position of *Eh*. Arrows indicate regions of interest where PKCδ is phosphorylated at the *Eh*-goblet cell contact site. Scale bar 10 μM. **D.** Quantification of confocal images was performed by measuring the concentration of phosphorylated PKCδ within the host cell in the immediate contact site by *Eh*. **E**. LS174T cells were pretreated with either the PI3K inhibitor wortmannin or PKC inhibitor bisindolylmaleimide I and stimulated with WT*Eh* secreted components over a 15 minute period. *** p <0.001, **p <0.01.

Previous studies have shown that mucin secretion from goblet cells is highly dependent on PKC [29, 30]. We observed a similar pattern where treatment with the broad spectrum PKC inhibitor bisindolylmaleimide I (BIM1; specific for PKC-α,β_i_,β_ii_,γ,δ,ε) markedly inhibited WT*Eh*-induced mucin secretion (Fig 5A). To initially screen for the specific PKC isoform responsible for mucin release, we tested three PKC inhibitors with varying IC_50_: rottlerin (indirectly inhibits PKCδ), bisindolylmaleimide IX (specific for PKC-α,β_i_,β_ii_,γ,ε) and Gö6983 (specific for PKC-α,β_i_,β_ii_,γ,δ,ζ) which mimic the broad action of BIM1. As bisindolylmaleimide IX failed to inhibit *Eh*CP5-dependent mucin secretion this ruled out PKC-α,β_i_,β_ii_,γ,ε suggesting that PKCδ or PKCζ might be the critical isoforms involved in mucin secretion. However, due to the potent effect of rottlerin on inhibiting mucin secretion, PKCδ was selected as the likely isoform responsible for secretion. To confirm this, western blot analysis of Phospho-PI3K and Phospho-PKCδ was also performed using WT*Eh* SC and recombinant *Eh*CP5 (Fig 5B) that confirmed these agonists potently activated these kinases. Confocal microscopy was used to image the interface between goblet cells and *Eh* with a phospho-PKCδ antibody (Fig 5C). Much like PI3K, only WT*Eh* induced the phosphorylation and activation of PKCδ at the contact site, whereas *Eh*CP5^-^ had no significant effect (Fig 5D). Live cell imaging of EGFP-PKCδ was also performed to track PKCδ translocation from the cytoplasm to the plasma membrane. Following contact with live WT*Eh* (blue) at the apical surface of the host cell, PKCδ (Green) can be seen associating with both the plasma membrane and intracellular vesicles indicative of activation (S1 Movie). This translocation of PKCδ did not occur in goblet cells contacted with live *Eh*CP5- (S2 Movie) as PKCδ can be observed only in the cytoplasm. Further, if the ATP-binding site within PKCδ is mutated (K376R) to produce a dominant negative kinase, live WT*Eh* (White) does not induce translocation of PKCδ (Green) to the plasma membrane (S3 Movie). Predictably, if goblet cells are stimulated with the PKC agonist PMA, PKCδ (Green) rapidly translocates from the cytoplasm to the plasma membrane (S4 Movie). Additionally, mucin positive intracellular granules (Red) can be observed associating with the plasma membrane after activation of PKCδ with PMA. To assess if PKCδ activation was a result of PI3K or vice/versa, cells were pretreated with either wortmannin or BIM1, subsequently exposed to *Eh* and the phosphorylation status of PI3K/PKCδ assessed by western blot (Fig 5E). As shown, WT*Eh* SC phosphorylated both PI3K and PKCδ in a time-dependent fashion. Pretreatment with wortmannin blocked downstream activation of PKCδ in response to WT*Eh*SC suggesting that PI3K acts upstream of PKCδ.

### *Eh*CP5 signaling results in activation of MARCKS

**Fig 6:**
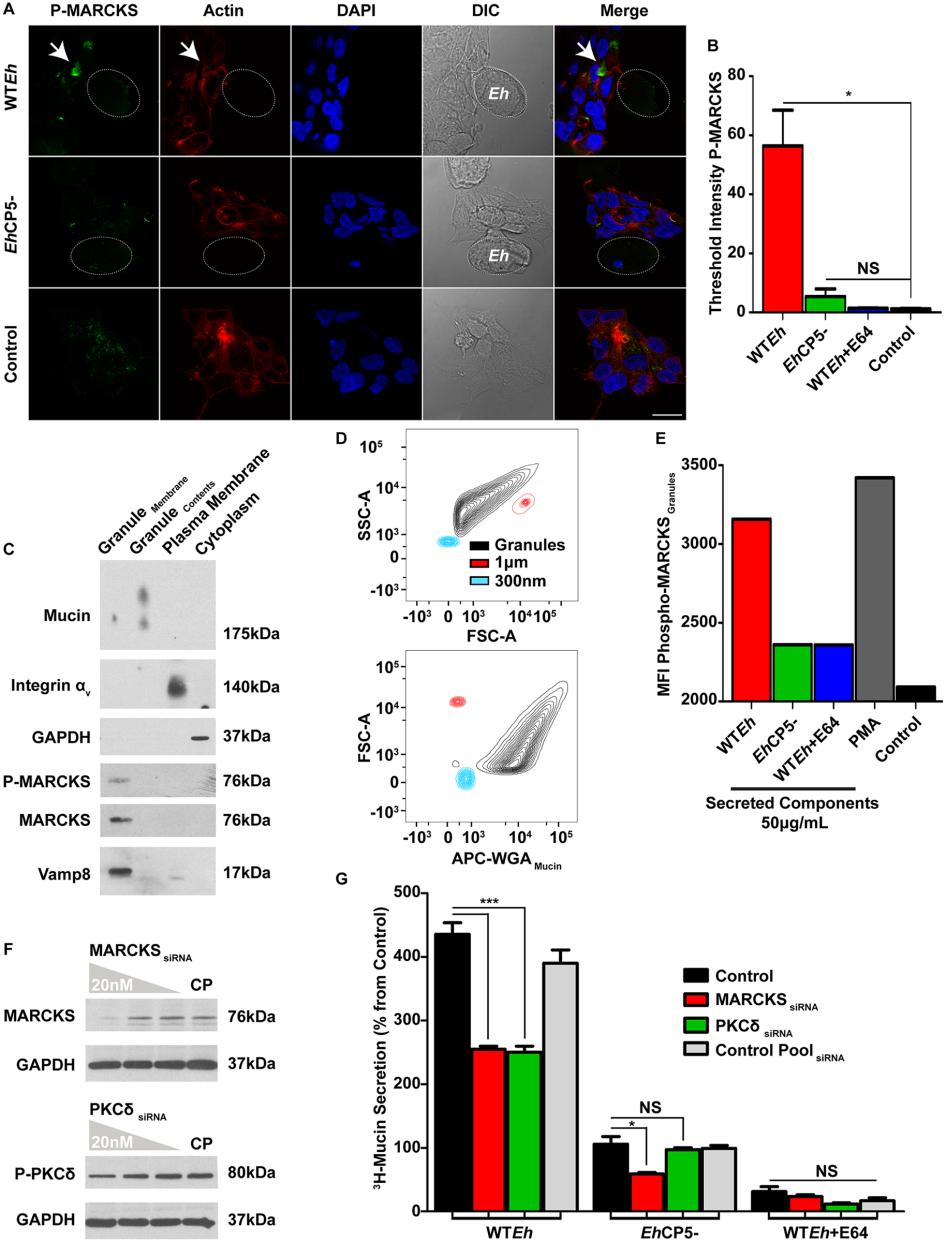
*Eh*CP5 signaling results in activation of MARCKS. **A.** LS174T cells were infected with live WT*Eh* or *Eh*CP5- for 15 minutes and subsequently immunostained for phospho-MARCKS and actin. Arrows indicate intracellular phospho-MARCKS in cells that are contacted by live WT*Eh*. The dotted white line indicates the position of *Eh*. Arrows indicate key regions of the cell that contains activated intracellular MARCKS that is phosphorylated. Scale bar 10 μm. **B.** Quantification of confocal images was performed by measuring the concentration of threshold phosphorylated PKCδ staining within the host cell in the immediate contact site by *Eh*. **C.** Mucin granules were extracted and purified from LS174T cells previously stimulated with 50μg/mL-secreted components from WT*Eh* along with subcellular fractionation of both the plasma membrane and cytoplasm. The following markers were used: Mucin for granule contents, Vamp8 for granule membrane, Integrin αv for the plasma membrane and GADPH for the cytoplasm. **D.** The gating strategy and calibration controls for flow cytometry of mucin granules using markers of known sizes 300nm (Blue), 1μm (Red) and presence of mucin within the granule as visualized with the lectin WGA (Black). **E.** Mucin granules were extracted from monolayers stimulated with 50μg/mL-secreted components from WT*Eh*, *Eh*CP5^-^, WT*Eh*+E64 or PMA (1μM) for 20 minutes and analyzed by flow cytometry using the gating strategy shown in 5D. Mean fluorescence intensity was measured from PE-Phospho-MARCKS whereby PMA served as the positive control. **F.** siRNA was used to disrupt MARCKS and PKCδ protein expression at concentrations of 20, 5, 1nM and compared to 20nM non-targeting control pool (CP). GAPDH serves as a loading control. **G.** ^3^H-glucoasmine metabolically labeled LS174T cells were transfected with siRNA against MARCKS, PKCδ or control pool and assayed for mucin secretion in response to live *Eh*. Control cells were treated with the transfection reagent in the absence of siRNA. *** p <0.001, *p <0.05.

Given the requirement for PKCδ activation in facilitating mucin secretion, the target of this phosphorylation event was interrogated. Within the airway epithelium, MARCKS has emerged as a key modulator driving exocytosis of mucin in goblet cells [31]. The ability of *Eh* to specifically activate MARCKS in a CP5-dependent manner was assessed by confocal microscopy in LS174T cells. As shown, WT*Eh* induced the phosphorylation of MARCKS and translocation to the cytoplasmic compartment, hallmarks in MARCKS facilitating mucin secretion in goblet cells (Fig 6A). *Eh*CP5^-^ did not induce the phosphorylation of MARKCS and localization similar to resting cells. Quantification of confocal images revealed phosphorylation of MARCKS was specific to WT*Eh* (Fig 6B). To confirm MARCKS was associating with mucin granules within the cytoplasm of LS174T cells, mucin granules were isolated and subsequently verified by western blot using known markers (Fig 6C). To quantify the population of mucin granules that contain phosphorylated MARCKS following exposure to WT*Eh* SC, purified mucin granules were analyzed by flow cytometry. FSC and SSC gates were established using latex calibration beads ranging from 300 nm (Blue) to 1 μm (Red); mucin granules (Black) are known to be approximately 1μm in diameter. Presence of glycosylated mucin was detected using a wheat-germ agglutinin (WGA) lectin and MFI of Phospho-MARCKS-PE was determined from WGA + granules (Fig 6D). WT*Eh* SC increased the activation state of MARCKS comparably to the positive control and potent mucin secretagogue PMA whereas *Eh*CP5^-^ SC or WT*Eh* + E64 did not (Fig 6E). The importance of MARCKS and PKCδ in facilitating mucin secretion was next assessed by siRNA knockdown. Specific siRNA for MARCKS and PKCδ was transfected at a range of 20nM-1nM where 20nM resulted in 83% and 41% knockdown respectively as measured by densitometry (Fig 6F). siRNA silencing of MARCKS and PKCδ both resulted in a similar decrease in WT*Eh*-induced mucin secretion as compared to non-targeting control pool siRNA (Fig 6G). With the exception of MARCKS knockdown on *Eh*CP5- induced mucin secretion, PKCδ nor any siRNA with WT*Eh*+E64 significantly hindered secretion of mucin.

### *Eh*CP5-αvβ3 signaling cascade is activated in an ex vivo model

**Fig 7:**
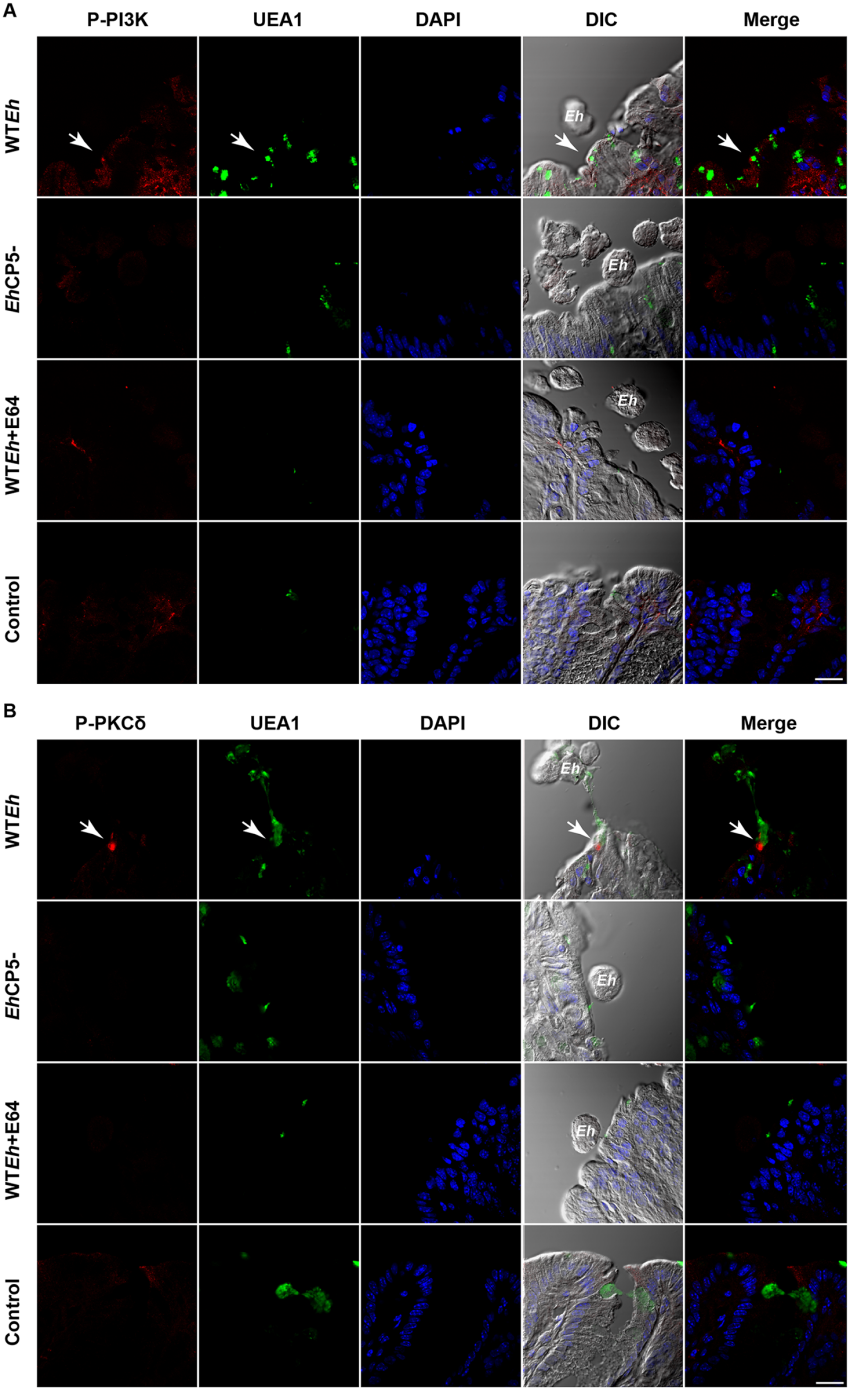
*Eh*CP5-αvβ3 signaling cascade is activated in an ex vivo model of *Eh* infection. Colonic explants were infected ex vivo with live WT*Eh*, *Eh*CP5^-^ or WT*Eh*+E64 for 15 minutes and immunostained for **A,** phospho-PI3K or **B,** PKCδ. Arrows indicate sites in the colonic explants that show activation of each respective kinase. UEA1 was used to detect secreted and goblet cell resident mucin and DIC were used to detect live *Eh* that contacted the epithelial cell surface. Arrows indicate critical sites of contact in which PI3K and PKCδ were activated. Scale bar 20 μm.

Initial studies using the colonic loop model of *Eh* infection proved unsuccessful due to the inability of *Eh* to intimately interact with the epithelium. This was a result of the adherent and/or increased mucus secretion together with water secretion following inoculation. Thus, an *ex vivo* model of *Eh* infection was used to assess the activation state of PI3K and PKCδ by confocal microscopy. To do this, colonic segments were washed with PBS to remove the adherent mucus layer and *Eh* was placed in intimate contact with the epithelium. Following infection for 15 minutes, WT*Eh* induced phosphorylated PI3K at the area around the contact site in epithelial cells including UEA1+ mucin goblet cells (Fig 7A). In *Eh*CP5- and WT*Eh*+E64 the global increase of phosphorylated PI3K was not observed in the epithelium despite several *Eh* within the vicinity. More strikingly, WT*Eh* induced UEA1+ mucin secretion from goblet cells that had strong phosphorylation of PKCδ resulting in *Eh* being repelled by mucus secretion (Fig 7B). Thick strands of UEA1+ mucus can be seen from a single goblet cell stuck to *Eh* in the lumen. No phosphorylation of PKCδ was observed in the epithelium of *Eh*CP5- or WT*Eh*+E64 infected tissues. These finding corroborate the *in vitro* findings that PI3K and PKCδ are acted upon to induce mucin secretion specifically by CP5 during *Eh* infection.

## Discussion

The current work presents a mechanism by which the virulence factor *Eh*CP5 present on the parasite surface and in secreted form can couple to αvβ3 integrin host receptor on goblet cells to induce hypersecretion of mucin. This highlights a critical yet overlooked aspect of *Eh* pathogenesis where hypersecretion can lead to depletion of mucin stores within goblet cells rendering the surface epithelium susceptible to *Eh* contact and subsequent invasion. We have previously characterized that *Eh* induces the secretion of glycosylated MUC2 mucin from LS174T cells [14]. Colonic mucin is protective by inhibiting adherence of *Eh* to host cells, a function that is abolished if O-linked glycosylation is inhibited [19]. In the present study, we interrogated the critical signaling cascades responsible for mucin hypersecretion and identified the putative virulence factor as *Eh*CP5 upon contact with goblet cells.

We have previously reported that *Eh*CP5 interact with αvβ3 integrin on colonic cells to stimulate a pro-inflammatory response mediated by NF-κB [21]. Whereas the pro-inflammatory response at the gene level was induced by the pro-form of CP5 irrespective of activity, hypersecretion of mucin was dependent on enzymatically active *Eh*CP5. Although it has been reported that activation of NF-κB can up regulate MUC2 gene expression, at the time points and MOI tested here this was not the case [32]. Various host intrinsic factors have been shown to up-regulate mucin gene expression and induce secretion. Specifically, pro-inflammatory cytokines such as IL-4, IL-6 and TNF-α have been implicated to induce secretion [33,34]. Even though the signaling cascades that govern these cytokines have not been worked out in the context of mucin secretion, all of these cytokines activate PI3K pathways. We hypothesize that activation of PI3K is likely conserved for its ability to cause mucin secretion, and various receptors and ligands upstream could likely be implicated by the work presented here. Indeed in other systems, PI3K has been shown to have a critical role for facilitating exocytosis [35]. Additionally, the presence of highly viscous mucinous secretions by various adenocarinomas and neoplasms could potentially be explained by alterations in PTEN or point mutations resulting in a gain of function within PIK3CA [36]. We showed here that activation of PI3K and production of PIP3 drives exocytosis of mucin, an event that is largely opposed in somatic cells by the actions of PTEN. Several kinases implicated in the present study such as PDK1 and FAK were determined to have a role in mucin secretion constitutively and irrespective of the agonist used. This could serve as the foundation for understanding a wide array of mucin secretion events during diseased states and shed light on the understudied mechanisms of how the mucus barrier is replenished to maintain homeostasis.

Perhaps the best understood mechanism in which an agonist induces mucin secretion is PGE_2_. PGE_2_ acting on EP1 receptors was shown to synergize the sensitivity of EP4 via a calcium dependent mechanism to generate cAMP [37]. While cAMP can induce mucin exocytosis, this is generally much slower (8–12 h) secretion than what is observed by PKC-dependent mechanisms [38]. Interestingly, *Eh* has a COX-like enzyme and in the presence of arachidonic acid can generate bioactive PGE_2_ [39]. It is plausible that the residual mucin secretion observed in CP5- deficient *Eh* or those lacking cysteine protease activity is a result of *Eh*-PGE_2_, since the signaling cascades implicated here have no overlap with that of EP receptors. Phorbol-esters, of which PKCδ is responsive, have long been known to be a potent and fast acting mucin secretagogue. It appears PKC is able to facilitate mucin exocytosis independent of calcium as chelation of intracellular calcium still allows for PKC-dependent secretion [37]. Additionally, Ca2^+^ ionophores seem to induce secretion via a protein kinase-independent mechanism. Given that PKCδ does not require calcium for its activation, our findings are in line with a calcium-independent PKC isoform facilitating mucin exocytosis. Additionally, no difference was observed in intracellular calcium between WT*Eh* and those that lacked CP5 or cysteine protease activity. While this does not completely exclude calcium involvement in facilitating mucin secretion, it remains to be investigated how this may occur and if calcium is utilized by the exocytosis machinery. Indeed, it is possible that there exist other mechanisms during *Eh* infection besides the *Eh*CP5-αvβ3 signal cascade however the contribution to mucin secretion is minimal.

The involvement of MARCKS in associating with mucin granules that were specifically targeted by *Eh*CP5 signaling provides valuable insight into the mechanism of exocytosis within goblet cells. In a mouse model of asthma, peptide inhibition of MARCKS was shown to inhibit mucus hypersecretion and alleviate disease within the airway [40]. MARCKS is a substrate for various PKC isoforms and has been shown to directly modulate actin dynamics at the plasma membrane [41,42]. In airway goblet cells, the activation and phosphorylation state of MARCKS dictates trafficking of mucin granules through the contractile actin cytoskeleton [31]. Although cortical actin was long believed to be a physical barrier to inhibit vesicle exocytosis, it is now apparent granules traffic along actin filaments and polymerization is critical for exocytosis. In the present study, live *Eh* induced F-actin at the contact site within the vicinity of phosphorylated PKCδ. The organizational arrangement of actin at the site of contact likely stems from Src kinase activation at the *Eh*CP5-αvβ3 intercellular junction. Src kinases have been implicated in other systems to modulate de novo actin polymerization during exocytosis [43].

It is possible PKCδ targets other components of the trafficking and exocytosis pathways to facilitate mucin secretion in goblet cells. Of particular interest are members of the Soluble NSF Attachment Protein Receptor (SNARE) family of exocytosis proteins, particularly SNAP23/25 and MUNC18. In neurons, SNAP25 is the predominant isoform expressed on the plasma membrane and utilized to facilitate exocytosis of neurotransmitters. It is well established that SNAP25 is a target of PKC phosphorylation at Ser187, an event that can be driven by phorbol-ester stimulation [44]. Although this appears to be mediated by conventional PKC isoforms (α, β), this is not surprising given the high calcium dependence of synapses. In non-neuronal secretory cells such as mast cells, SNAP23 is commonly utilized for exocytosis and is the target of phosphorylation at Ser95 and Ser120 [45]. Phosphorylation double mutants have been shown to have an impaired ability to undergo exocytosis in response to stimuli however this event appears to be driven by IκB kinase 2 [46]. The SNARE chaperone MUNC18 is responsible for assisting in vesicle fusion by controlling interactions of various SNARE components. In chromaffin cells, MUNC18 is phosphorylated resulting in a change in vesicle dynamics and kinetics of exocytosis [47]. Additionally, pancreatic beta cells have been shown to utilize PKCδ in facilitating insulin secretion [48]. Here, PKCδ phosphorylates Munc18-1 to allow insulin exocytosis to occur, an event that is drastically hindered in PKCδ null mice. Indeed, further examination of the core SNARE complex and trafficking members are necessary to discern how goblet cells release mucin granules both constitutively and in response to pathogenic stimuli.

Pathogens use a variety of mechanisms to induce mucin secretion. *Listeria monocytogenes* induces mucin exocytosis through the pore-forming toxin listeriolysin-O, a factor that is dependent on the toxins internalization into the host cell and independent of bacteria entry [49]. However, the signaling cascades that govern this secretion remain elusive. Elastases have been demonstrated to be mucin secretagogues in *Pseudomonas aeruginosa* and the virulence factor Pic in Enterobacteriaceae spp [50,51]. These serine proteinases differ, as elastase requires protease activity to induce secretion whereas Pic does not. Here we show that *Eh*CP5 requires protease activity to evoke mucin secretion from goblet cells. The importance of proteinase activity in establishing infection has been noted by the ability of *Eh* to induce cytolysis of host cells in culture [52]. Additionally, *Eh* lacking CP activity produce smaller liver abscesses in the gerbil model of amebiasis [53]. The ability for *Eh* to disseminate to soft organs appear to be dependent on *Eh*CP5 as cells lacking this proteinase does not induce liver abscess formation [52]. *Eh*CP5 is emerging as a multifunctional enzyme that plays critical roles in the pathogenesis of intestinal amebiasis by first cleaving the C-terminus of MUC2 to dissolve the mucus layer and second, to bind αvβ3 integrins receptors on goblet cells to trigger mucus hypersecretion and pro-inflammatory responses.

## Materials and Methods

### Cell culture

Human adenocarcinoma colonic cells LS174T (ATCC) was cultured in Eagle’s minimum essential media (Hyclone) containing 20mM HEPES, 100U/mL penicillin/streptomycin sulfate with 10% fetal bovine serum. Cells were maintained at 37°C with 5% CO_2_ in T75 flasks. Media was replaced every 3 days and cells harvested with 0.25% Trypsin/EDTA (Invitrogen). For metabolic labeling of mucin, LS174T were seeded in 24 well dishes at 5x10^4^ cells/well in triplicate and allowed to reach 90% confluence. For biochemical assays to assess phosphorylation cells were seeded in 6 well dishes at 8 x 10^5^ cells/well, allowed to reach 70% confluence and serum starved 12 h prior to assay.

### Cultivation and harvesting of *E*. *histolytica*

*Eh* (HM1:IMSS; ATCC) and *Eh*CP5^-^ (a gift from D. Mirelman; Weizmann Institute of Science) trophozoites were cultured in TYI-S-3 medium containing 100U/mL penicillin/streptomycin sulfate at 37°C in sealed borosilicate glass tubes under axenic conditions [52]. *Eh*CP5- was generated by transfecting pSA8, which contains an antisense cassette for the *Eh*CP5 gene along with neomycin resistance. AmoebaporeA antisense *Eh* were also included as a vector control for silencing *Eh*CP5- as this strain lacks AmoebaporeA. In experiments where cysteine protease activity was inhibited, 100uM E64 was included in the culture media overnight [53]. After 72 h, *Eh* trophozoites were harvested by chilling 9 minutes on ice, subsequently pelleted at 200 *x*g, and washed twice with PBS. To maintain high virulence, *Eh* trophozoites were regularly passaged through the liver of gerbils as described elsewhere [54]. For the preparation of *Eh* secreted components, 1 x 10^7^ *Eh*/mL were suspended in Hanks balanced salt solution and incubated at 37°C for 2 h. Live *Eh* were subsequently cleared from the preparation by centrifugation at 10,000 *x*g and secreted components stored at -80°C. Protein content was assessed by BCA assay (Thermo Fischer).

### Reagents

All reagents unless otherwise specified were from Sigma-Aldrich. Wortmannin, Ly294002, rottlerin, bisindolylmaleimide i, bisindolylmaleimide ix, Gö6983 were from Cayman Chemical. P11, GRGDSP, GRADSP, αvβ3 function blocking antibody (LM609) were from EMD Millipore. PP1, PP2, PP3 were from Calbiochem. LDH assay was from Promega. Phospho-MARCKS Ser152/156 (Cat#2741), MARCKS (Cat#5607), Phospho-PI3K (Cat#4228), p85 PI3K (Cat#4257), Phospho-PKCδ (Cat#2055), PKCd (Cat#9616) antibodies were from Cell Signaling Technology. FITC-PIP3 antibody (Cat#G345) was from Echelon. All secondary antibodies and isotype controls were from Life Technologies.

### αvβ3 binding ELISA

Adhesive proteins were coated on ELISA plates using 100mm Bicarbonate/carbonate coating buffer overnight at 4°C. Various concentrations of adhesive proteins were coated with 10ug being optimal. Following blocking with PBS+1% BSA for 1 hour and washing with PBS+0.05% Tween thrice, individual wells were incubated with 50ng/well recombinant αvβ3 (EMD Millipore) in blocking buffer for 1 hour at 37°C. Plates were washed three times with wash buffer followed by 500ng/mL αv detection antibody (R&D Systems Cat#MAB1219), three washes then 50ng/mL mouse IgG-HRP. Detection of bound αvβ3 was visualized by TMB substrate and read at Abs450.

### Flow cytometry

1 x 10^6^ cells/well were seeded in 6 well dishes 24 h prior to infection with *Eh*. Following infection for 15 minutes, cells were washed twice in PBS and trypsinized to obtain a cell suspension. After centrifugation at 200 *x*g, cells were suspended in ice-cold methanol for fixation and subsequently stained as outlined above however FACS buffer (2% calf serum, 1mM EDTA in calcium/magnesium free PBS) was employed in place of PBS. Prior to flow cytometry analysis, cells were strained through a 40um filter. Unstained LS174T cells were used to establish forward and side scatter gates whereby *Eh* were completely excluded. This was confirmed by DAPI positive cells as *Eh* are not stained by this dye. A positive result was inferred from cells that demonstrated an increase in mean-fluorescence intensity over isotype controls. In all cases, a minimum of 20,000 cells was counted per condition. Data was analyzed in FlowJo.

### Confocal microscopy

1x10^6^ cells/well were seeded on 5cm^2^ No. 1.5 glass coverslips 24 h prior to infection with *Eh*. Prior to the assay, cells were washed twice with PBS and then challenged with 2x10^5^ *Eh*/well (MOI: 0.2). For PIP3 staining, coverslips were transferred to ice-cold methanol for 5 minutes followed by extensive washing with PBS. For Phospho-PI3K, PKCδ, MARCKS, FAK and SRC coverslips were fixed in 3.7% paraformaldehyde for 20 minutes, washed with PBS and permeabilized with PBS containing 0.35% Triton. Coverslips were then blocked with 5% normal donkey serum and incubated overnight with primary antibodies in a humidified chamber at 4°C. The following day, coverslips were washed with PBS containing 0.1% Tween and incubated at RT with fluorescent secondary antibodies, phalloidin and DAPI (Life Technologies). Coverslips were mounted with Fluorosave reagent (Calbriochem) and visualized on an Olympus FV1000 scanning confocal inverted microscope. For live cell imaging experiments, cells were transfected in optiMEM with pEGFP-huPKCdelta (gift from P. Blumberg), pEGFP-huPKCdeltaK376R-DN, or the MUC2 reporter pmRuby2-MUC2CK using Lipofectamine 2000. Images were processed in Adobe Photoshop CS5. Quantification and analysis was done in FIJI (ImageJ). To determine the abundance of each protein at the *Eh*-goblet cell interface, *Eh* was traced using DIC and the area 150% larger was considered the immediate contact site with the area *Eh* occupies being excluded. To limit this area to just where the host cells occupied, the host cell boundary was also traced out and only where the aforementioned gates overlaid was considered the contact site of *Eh*. For all quantification with the exception of MARCKS, raw integrated density values were subtracted from background and subsequently divided by the size of the gate to be expressed as average mean intensity. Since Phospho-MARCKS was either present or absent following *Eh* contact, the same gating strategy was used as above however, the area 200% larger was included and threshold values of 160/255 were reported.

### PIP3 quantification

1 x 10^6^ cells/well were seeded in 6 well dishes and allowed to reach 70% confluence in complete media. Cells were then labeled with 10uCi/mL ^3^H-Myo-inositol in DMEM lacking inositol and dialyzed FBS for 24 h. After incorporation of ^3^H into phosphoinositols, cells were washed twice with PBS and incubated for 20 minutes with serum-free EMEM containing 50μg/mL *Eh* secreted components or live *Eh* (2 x 10^5^ *Eh*/well). Acidic lipids, of which PIP3 is present, were then extracted under organic conditions as previously described [55]. Briefly, cells were collected in cold 0.5M TCA and centrifuged at 4000 *x*g. The pellet was then washed once with 5%TCA/1mM EDTA and once with MeOH: CHCl_3_ (2:1) after a 15-minute incubation on ice. Acidic lipids were then extracted with a 80:40:1 ratio of MeOH:CHCl_3_:12MHCl, incubated for 15 minutes and pellet discarded after centrifugation. Phase spilt was performed on the supernatant using acidified CHCl_3_ and after centrifuging the organic layer was dried. Pull-down was then performed on dried lipid in PBS with 2μg of recombinant GST-GRP1-PH and 50uL of HiBind Glutathione agarose [56]. After washing twice with PBS and subsequent centrifugation at 4000 *x*g, ^3^H-PIP3 was counted directly by scintillation.

### Quantification and validation of mucin secretion

Mucins from LS174T cells were metabolically labeled with 2uCi/mL of ^3^H-glucosamine. After 24 h, cells were washed once with PBS and twice with serum-free EMEM. For kinase inhibitor studies, cells were pretreated with various inhibitors for up to 30 minutes, washed once in EMEM and subsequently stimulated with *Eh* in the presence of each respective inhibitor. For function blocking antibody and small peptide inhibitor studies, cells were pretreated for 5 minutes and then stimulated with *Eh* without washing. Unless otherwise stated, all secretion experiments were carried out for 120 minutes at which time 80% of the supernatant was loaded into scintillation vials for counting (Beckman Coulter). In all experiments where inhibitors or blocking peptides were used, a dose response was performed to determine the optimal dose for inhibition of mucin secretion. Unless otherwise stated, control refers to baseline mucin secretion with just the vehicle. For quantification of ^3^H-mucin secretion, up to 6 wells/condition were pooled for analysis by Sepharose 4B chromatography as previously described [13]. Briefly, secreted ^3^H-labelled mucin in the cell culture supernatants were precipitated with an equal volume of 10%TCA/1%PTA at 4°C overnight and subsequently spun at 4000 *x*g and suspended to neutral pH in 0.01M Tris-base containing 5% glycerol. The ^3^H-mucin was then loaded onto a 100mL Sepharose 4B chromatography column containing 0.01M Tris-base and 1mL fractions collected for scintillation counting.

### SDS-PAGE western blot

Following the assay, cells were washed three times with ice-cold PBS and lysed in a buffer composed of 20mM HEPES, 150mM NaCl, 1mM EDTA, 1% NP-40, 10uM E64 and a protease inhibitor cocktail (Roche). Samples were then cleared by centrifuging at 14,000 *x*g and supernatants quantified for protein content by BCA assay. Prior to analysis by SDS-PAGE, Laemmli sample buffer containing 5% BME was added to samples (20μg/well) and boiled for 5 minutes. Samples were resolved on 10% polyacrylamide gels, wet transferred to 0.2um nitrocellulose and blocked with either 5% skim milk or 3% BSA (Phospho-proteins). Primary antibodies diluted in PBS containing 0.1% Tween and 5% BSA were incubated overnight with blots at 4°C. Following extensive washing, blots were incubated at RT for 2 h with secondary antibodies coupled to HRP and developed using ChemiLucent ECL detection (EMD Millipore, Billerica, MA) on film.

### Recombinant CP5 production

Recombinant CP5 was generated in BL21 *E*. *coli* under denaturing conditions as described elsewhere [57]. Briefly, BL21 cells harboring the cDNA of CP5 (aa14-317) in pJC45 were grown to an OD of 0.4 induced with 1mM IPTG and allowed to grow for an additional 4 h. Although some rCP5 resided in the soluble fraction, the cells were lysed under denaturing conditions using a buffer containing first 6M GuCl then 8M Urea. Following pull-down overnight with Ni/NTA agarose, beads were washed extensively on a chromatography column with a phosphate buffer containing 20mM imidazole. Recombinant protein was eluded with 300mM imidazole and subsequently added drop wise into 1000-fold volume of refolding buffer containing 5mM GSSG and GSH. Recombinant CP5 was then salted-out with ammonium sulfate (35% w/v) and stored at -80°C. Prior to the experiment, rCP5 was activated for 1 h at 37°C in a buffer containing 20mM Tris, 2% SDS and 1mM DTT. Protease activity was measuring using the colorimetric Z-Arg-Arg-pNA assay as described previously [56]. Recombinant CP5 purity was routinely assessed by silver stain where two bands at 35 and 25kDa make up >90% of the protein recovered by this method.

### Ethics statement

The Health Sciences Animal Care Committee from the University of Calgary, have examined the animal care and treatment protocol (M08123) and approved the experimental procedures proposed and certifies with the applicant that the care and treatment of animals used was in accordance with the principles outlined in the most recent policies on the “Guide to the Care and Use of Experimental Animals” by The Canadian Council on Animal Care.

### Ex vivo infection with *Eh*

Colonic explants from mice were infected with live *Eh* as previously described [58]. Briefly, the distal portion of the colon from SV129 mice was flushed extensively with PBS and subsequently cut longitudinally and further into 10mm^2^ segments. These colonic explants were pinned into transwells with the mucosal surface facing up. Infection was carried out by laying 1x10^5^ *Eh*/well in HBSS, infected for 15 minutes and fixed in 3.7% paraformaldehyde. Segments were then processed for cyrostat by passing through a sucrose gradient, embedding in OCT and then sectioned at 10μm. Immunostaining was as described above and mucin was visualization using UEA1-FITC (Sigma).

### Statistics

Experiments presented are representative of at least three independent experiments. Statistical significance between two or more groups was assessed by one-way ANOVA whereby p<0.05 was considered significant. For comparison between two groups the Student’s t-test was used. For quantification of confocal images a minimum of 8 images were used per condition. Results presented in histograms are displayed as the mean with the standard error of the mean for error bars.

## Supporting Information

S1 Fig **A. *Eh*-induced cell death at 1 and 2 h post infection on LS174T confluent monolayers was assessed by LDH assay.** The percent cell death was extrapolated from lysing cells with triton and counting the total amount of LDH. **B**. Live, glutaraldehyde fixed or heat-killed *Eh* was added to LS174T goblet cells metabolically labeled with ^3^H-glucosamine and assessed for the ability to induce ^3^H-mucin secretion. **C**. Attachment of *Eh* to confluent LS174T monolayers was measured by CFSE labeling *Eh* following contact for 30 minutes. GFP fluorescence was then measured and compared to total amount of *Eh* placed per well. **D**. Western blot analysis of WT*Eh* secreted components and recombinant *Eh*CP5 as detected by a CP5 antibody. **E**. Relative cysteine protease activity of *Eh* secreted components as measured by calorimetric Z-Arg-Arg-pNA assay. **F.** qPCR for MUC2 mRNA following treatment with either PMA or WT*Eh*.***p<0.001.
(TIF)

S2 Fig **A. LS174T cells were infected with live WT*Eh*, *Eh*CP5** ^**-**^ **or WT*Eh*+E64 and supernatants assayed for presence of ATP with CellTitre Glo kit (Promega) at various time points. B.** Live WT*Eh*, *Eh*CP5^-^ or WT*Eh*+E64 were tested for the ability to induce calcium flux in Fluo4 loaded LS174T cells as measured by live cell confocal imaging. *Eh* was added at t = 50 and all conditions were spiked with calcium ionophore (A23187; 1μM) at t = 500.
(TIF)

S3 Fig Mucins from LS174T goblet cells metabolically labeled with ^3^H-glucosamine were treated with A, PI3K inhibitor Ly294002 (10μM) B, AKT inhibitor Triciribine (10μM) or C, PDK1 inhibitor GSK2334470 (10μM) and assayed for ^3^H-labeled mucin secretion after 2 h of infection. ***p <0.001, **p <0.01, *p <0.05.
(TIF)

S1 Movie LS174T cells were transfected with pEGFP-PKCδ using lipofectamine 2000 and infected 24 h later with WT*Eh* that were labeled with CellTracker Blue. (MOV)

S2 Movie LS174T cells were transfected with pEGFP-PKCδ using lipofectamine 2000 and infected 24 h later with *Eh*CP5- that were labeled with CellTracker Blue. (MOV)

S3 Movie LS174T cells were transfected with dominant negative pEGFP-PKCδ-K376R using lipofectamine 2000 and infected 24 h later with WT*Eh* that were labeled with CellMask Red (Pseudocolored white). (MOV)

S4 Movie LS174T cells were transfected with pEGFP-PKCδ and a mucin reporter construct (pmRuby2-MUC2CK; Red) using lipofectamine 2000. Nuclei were also stained using NucBlue. After 24 h, the cells were stimulated with 1μM PMA as a positive control for PKCδ activation.
(MOV)
