# Supplementary figures and images for: Entamoeba histolytica Cysteine Proteinase 5 Evokes Mucin Exocytosis from Colonic Goblet Cells via αvβ3 Integrin
Source: PLoS Pathog. 2016 Apr 13;12(4):e1005579. doi: 10.1371/journal.ppat.1005579 (PMC4830554; doi:10.1371/journal.ppat.1005579)

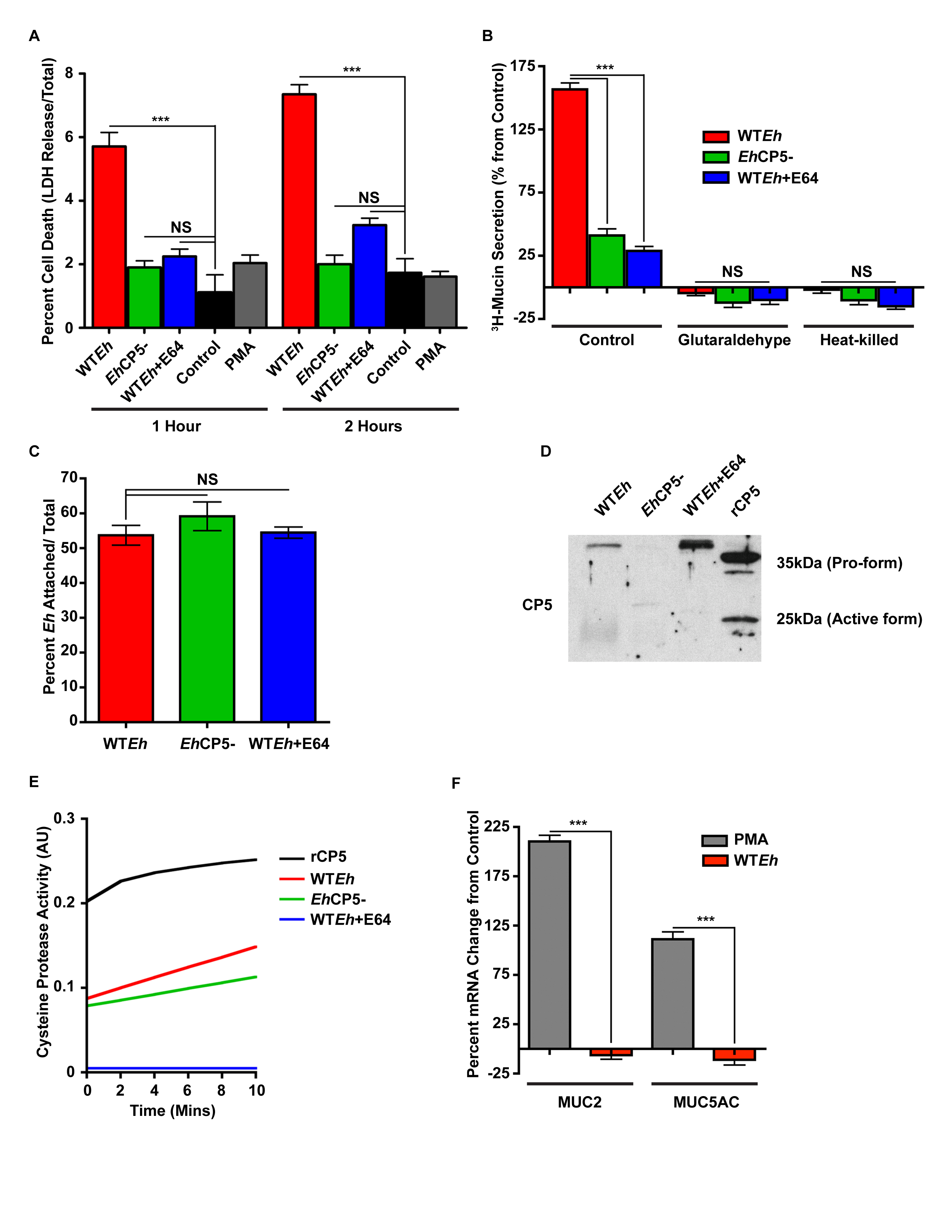

Supplement: S1 Fig — A. Eh-induced cell death at 1 and 2 h post infection on LS174T confluent monolayers was assessed by LDH assay. The percent cell death was extrapolated from lysing cells with triton and counting the total amount of LDH. B. Live, glutaraldehyde fixed or heat-killed Eh was added to LS174T goblet cells metabolically labeled with 3H-glucosamine and assessed for the ability to induce 3H-mucin secretion. C. Attachment of Eh to confluent LS174T monolayers was measured by CFSE labeling Eh following contact for 30 minutes. GFP fluorescence was then measured and compared to total amount of Eh placed per well. D. Western blot analysis of WTEh secreted components and recombinant EhCP5 as detected by a CP5 antibody. E. Relative cysteine protease activity of Eh secreted components as measured by calorimetric Z-Arg-Arg-pNA assay. F. qPCR for MUC2 mRNA following treatment with either PMA or WTEh.***p<0.001. (TIF) [file ppat.1005579.s001.tif]

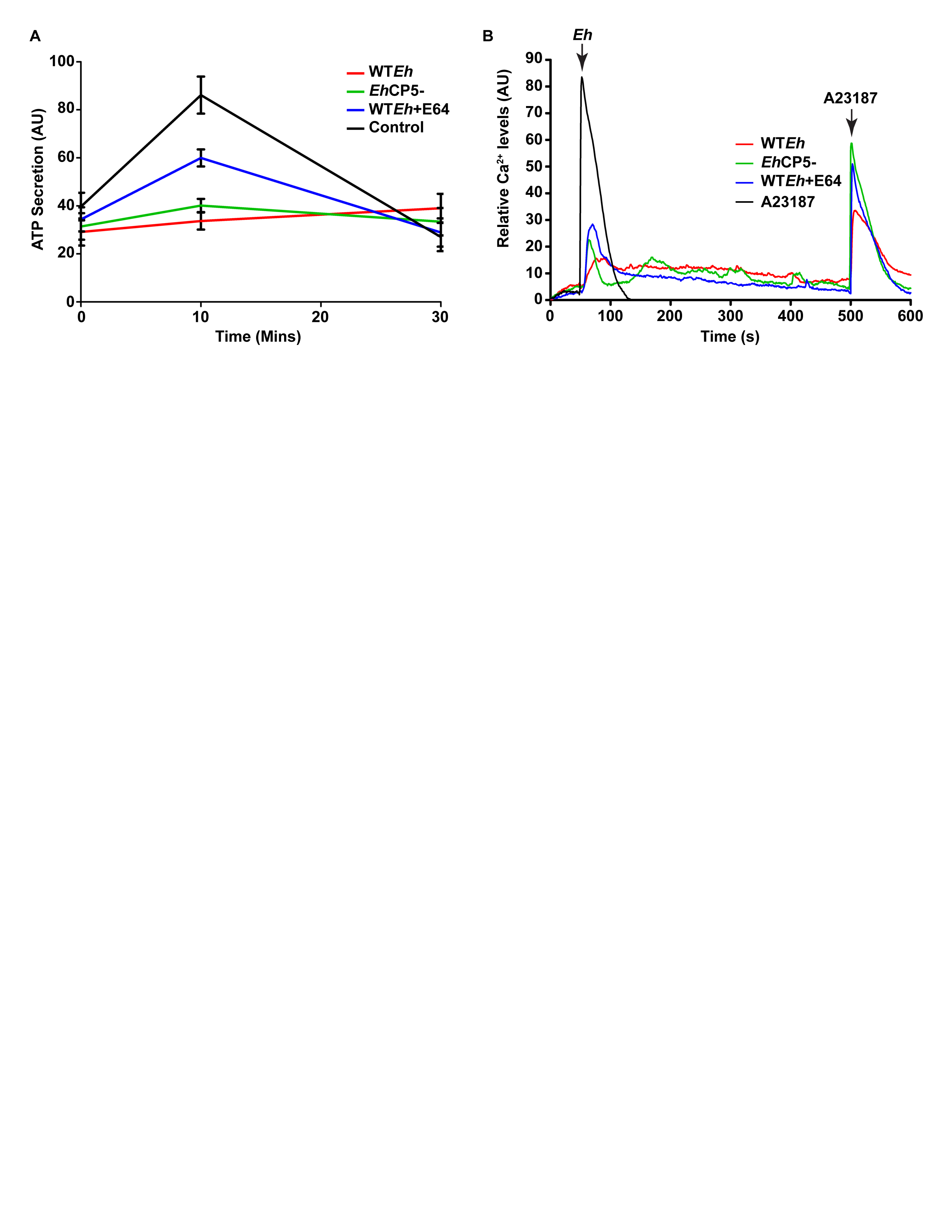

Supplement: S2 Fig — A. LS174T cells were infected with live WTEh, EhCP5 - or WTEh+E64 and supernatants assayed for presence of ATP with CellTitre Glo kit (Promega) at various time points. B. Live WTEh, EhCP5- or WTEh+E64 were tested for the ability to induce calcium flux in Fluo4 loaded LS174T cells as measured by live cell confocal imaging. Eh was added at t = 50 and all conditions were spiked with calcium ionophore (A23187; 1μM) at t = 500. (TIF) [file ppat.1005579.s002.tif]

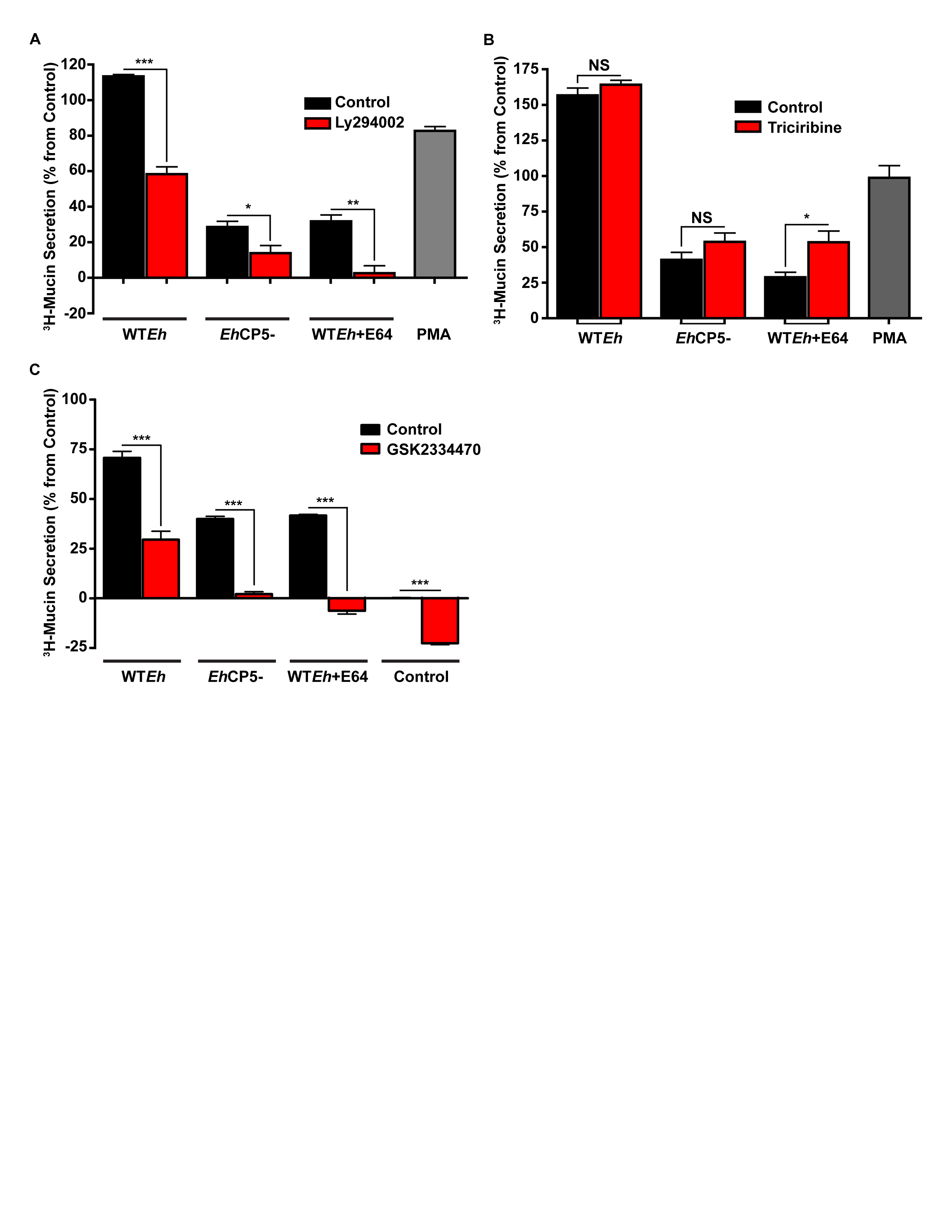

Supplement: S3 Fig — ***p <0.001, **p <0.01, *p <0.05. (TIF) [file ppat.1005579.s003.tif]
